# Research priorities for global food security under extreme events

**DOI:** 10.1016/j.oneear.2022.06.008

**Published:** 2022-07-15

**Authors:** Zia Mehrabi, Ruth Delzeit, Adriana Ignaciuk, Christian Levers, Ginni Braich, Kushank Bajaj, Araba Amo-Aidoo, Weston Anderson, Roland A. Balgah, Tim G. Benton, Martin M. Chari, Erle C. Ellis, Narcisse Z. Gahi, Franziska Gaupp, Lucas A. Garibaldi, James S. Gerber, Cecile M. Godde, Ingo Grass, Tobias Heimann, Mark Hirons, Gerrit Hoogenboom, Meha Jain, Dana James, David Makowski, Blessing Masamha, Sisi Meng, Sathaporn Monprapussorn, Daniel Müller, Andrew Nelson, Nathaniel K. Newlands, Frederik Noack, MaryLucy Oronje, Colin Raymond, Markus Reichstein, Loren H. Rieseberg, Jose M. Rodriguez-Llanes, Todd Rosenstock, Pedram Rowhani, Ali Sarhadi, Ralf Seppelt, Balsher S. Sidhu, Sieglinde Snapp, Tammara Soma, Adam H. Sparks, Louise Teh, Michelle Tigchelaar, Martha M. Vogel, Paul C. West, Hannah Wittman, Liangzhi You

**Affiliations:** 1 Department of Environmental Studies, University of Colorado, Boulder, CO, USA; 2 Mortenson Center in Global Engineering, University of Colorado Boulder, Boulder, CO, USA; 3 University of Basel, Basel, Switzerland; 4 Food and Agriculture Organization of the United Nations, Rome, Italy; 5 Department of Environmental Geography, Institute for Environmental Studies, Vrije Universiteit Amsterdam, Amsterdam, the Netherlands; 6 Institute for Resources, Environment and Sustainability, University of British Columbia, Vancouver, BC, Canada; 7 Kassel University, Department of Agricultural Engineering, Kassel University, 37213 Witzenhausen, Germany; 8 Kumasi Technical University, Department of Automotive and Agricultural Mechanization, P.O. Box 854, Kumasi, Ghana; 9 Earth System Science Interdisciplinary Center, University of Maryland, College Park, MD 20740, USA; 10 International Research Institute for Climate and Society, Columbia University, Palisades, NY 10964, USA; 11 College of Technology, The University of Bamenda, Bamenda, Cameroon; 12 Higher Institute of Agriculture and Rural Development, Bamenda University of Science and Technology – BUST, Bamenda, Cameroon; 13 Royal Institute of International Affairs, Chatham House, 10 St James Sq, London SW1Y 4LE, UK; 14 Risk & Vulnerability Science Centre, Faculty of Science & Agriculture, University of Fort Hare, Alice, South Africa; 15 Department of Geography & Environmental Systems, University of Maryland, Baltimore County, 1000 Hilltop Circle, Baltimore, MD 21250, USA; 16 Université Félix Houphouet-Boigny, Abidjan, Côte d’Ivoire; 17 International Institute for Applied Systems Analysis (IIASA), Schlossplatz 1, 2361 Laxenburg, Austria; 18 Potsdam Institute for Climate Impact Research (PIK), Telegrafenberg, 14473 Potsdam, Germany; 19 Universidad Nacional de Río Negro, Instituto de Investigaciones en Recursos Naturales, Agroecología y Desarrollo Rural, Río Negro, Argentina; 20 Consejo Nacional de Investigaciones Científicas y Técnicas, Instituto de Investigaciones en Recursos Naturales, Agroecología y Desarrollo Rural, Río Negro, Argentina; 21 Institute on the Environment, University of Minnesota, St. Paul, MN 55108, USA; 22 Agriculture and Food Business Unit, Commonwealth Scientific and Industrial Research Organisation, St Lucia, QLD, Australia; 23 Ecology of Tropical Agricultural Systems, Institute of Agricultural Sciences in the Tropics, University of Hohenheim, Stuttgart, Germany; 24 Kiel Institute for the World Economy (IfW), Kiel, Germany; 25 Environmental Change Institute, School of Geography and the Environment, University of Oxford, Oxford, UK; 26 Department of Agricultural and Biological Engineering, University of Florida, Gainesville, FL 32611, USA; 27 School for Environment and Sustainability, University of Michigan, Ann Arbor, MI, USA; 28 UMR MIA 518, Université Paris-Saclay, INRAE, AgroParisTech, Paris, France; 29 Human Sciences Research Council (HSRC), Africa Institute of South Africa (AISA), 134 Pretorius Street, Pretoria, Gauteng, South Africa; 30 Keough School of Global Affairs, University of Notre Dame, Notre Dame, IN, USA; 31 Department of Geography, Faculty of Social Sciences, Srinakharinwirot University, Bangkok, Thailand; 32 Leibniz Institute of Agricultural Development in Transition Economies (IAMO), Theodor-Lieser-Str. 2, 06120 Halle (Saale), Germany; 33 Faculty of Geo-Information Science and Earth Observation (ITC), University of Twente, Enschede, the Netherlands; 34 Agriculture and Agri-Food Canada, Science and Technology Branch, Summerland Research and Development Centre, Summerland, BC, Canada; 35 Food and Resource Economics Group, the University of British Columbia, Vancouver, BC, Canada; 36 Centre for Agriculture and Biosciences International (CABI), 673 Canary Bird, Limuru Road, Muthaiga, Nairobi, Kenya; 37 Jet Propulsion Laboratory/California Institute of Technology, Pasadena, CA, USA; 38 Max-Planck-Institute for Biogeochemistry, Jena, Germany; 39 Department of Botany and Biodiversity Research Centre, University of British Columbia, Vancouver, BC, Canada; 40 European Commission Joint Research Centre, Ispra, Italy; 41 The Alliance of Bioversity International and International Center for Tropical Agriculture, Rome, Italy; 42 Department of Geography, University of Sussex, Brighton, UK; 43 Lorenz Center, Department of Earth, Atmospheric and Planetary Sciences, Massachusetts Institute of Technology, Cambridge, MA, USA; 44 Helmholtz Institute for Environmental Research (UFZ), Leipzig, Germany; 45 Institute of Geoscience and Geography, Martin-Luther-University Halle-Wittenberg, Halle (Saale), Germany; 46 Department of Plant, Soil and Microbial Sciences, Center for Global Change and Earth Observations, Michigan State University, East Lansing, MI, USA; 47 School of Resource and Environmental Management, Simon Fraser University, Burnaby, BC, Canada; 48 Department of Primary Industries and Regional Development, Perth, WA 6000, Australia; 49 University of Southern Queensland, Centre for Crop Health, Toowoomba, QLD 4350, Australia; 50 Institute for the Oceans and Fisheries, University of British Columbia, Vancouver, BC, Canada; 51 Center for Ocean Solutions, Stanford University, Stanford, CA, USA; 52 Man and the Biosphere Programme, Division of Ecological and Earth Sciences, Natural Sciences Sector, UNESCO, Paris, France; 53 Department of Applied Economics, University of Minnesota, St. Paul, MN 55108, USA; 54 Project Drawdown, 3450 Sacramento Street, San Francisco, CA, USA; 55 International Food Policy Research Institute, Washington, DC, USA

## Abstract

Extreme events, such as those caused by climate change, economic or geopolitical shocks, and pest or disease epidemics, threaten global food security. The complexity of causation, as well as the myriad ways that an event, or a sequence of events, creates cascading and systemic impacts, poses significant challenges to food systems research and policy alike. To identify priority food security risks and research opportunities, we asked experts from a range of fields and geographies to describe key threats to global food security over the next two decades and to suggest key research questions and gaps on this topic. Here, we present a prioritization of threats to global food security from extreme events, as well as emerging research questions that highlight the conceptual and practical challenges that exist in designing, adopting, and governing resilient food systems. We hope that these findings help in directing research funding and resources toward food system transformations needed to help society tackle major food system risks and food insecurity under extreme events.

## Highlights

• We asked experts for top threats to global food security from extreme events
• We find unresolved governance challenges underpin many of the key threats
• We also asked experts for top outstanding research priorities on this topic
• Coordination to design, adopt, and govern resilient food systems is needed

## Science for society

Heat waves, floods, droughts, pest outbreaks and diseases, financial crises, and human conflicts are threatening the production and supply of food around the world. These extreme events are on the rise, and our ability to prepare for them seems limited. Multiple events occurring at the same time compound the problem. Research teams and policymakers are developing solutions to improve the resilience of food systems, but this is often done in isolation—tackling one problem at a time. In this article we bring together food system experts to identify the top threats over the next two decades and priority research questions to address them. We find that unresolved governance challenges in international relations underpin many of the key threats and that coordinated research is needed to help design and adopt systems of governance for food systems that are resilient to extreme events in the future.

## Graphical abstract

**Figure:**
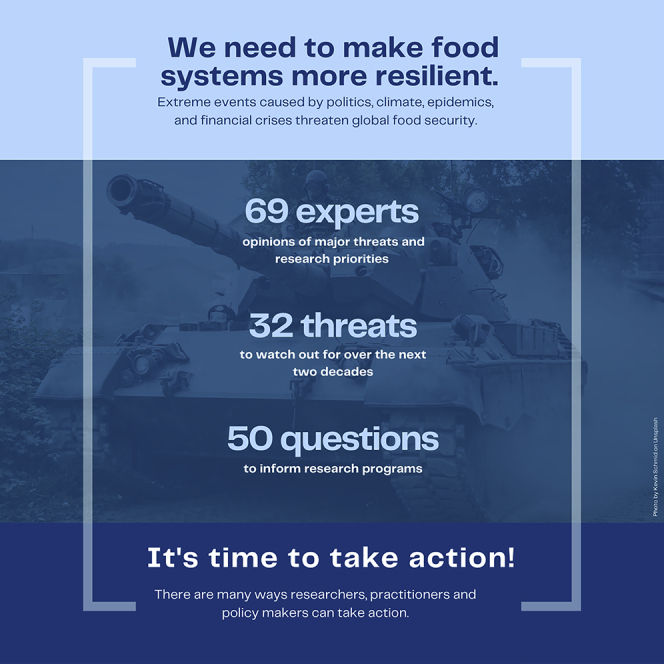

## Teaser

Extreme events threaten the production and supply of food around the world. They create cascading and systemic impacts posing significant challenges to food systems research and policy alike. However, research teams and policymakers are not tackling these connections and are developing solutions in isolation. We bring together experts to prioritize threats to global food security from extreme events as well as research. Our findings illustrate the importance of coordinated design, adoption, and governance of food systems for resilience.

## Introduction

Extreme events caused by climate change, economic or geopolitical shocks, and pest or disease epidemics can induce, spread, and prolong food insecurity.^1^^,^^2^ They do this by reducing farming and fisheries productivity, threatening subsistence, and disrupting food distribution and public service delivery. Extreme events can also drive increases in food prices and volatility, human migration, and political instability. These direct and indirect effects lead to reductions in the availability of, and access to, healthy and nutritious food.^1^, ^2^, ^3^, ^4^, ^5^, ^6^ The magnitude, extent, and complexity of the threats posed by extreme events to global food security can further create cascading and systemic impacts^7^^,^^8^ that are difficult to predict or plan and prepare for.

Despite the continued focus on research on extreme events and food security, many key areas remain disconnected. For example, there has been much research on extreme weather's impact on crop yields, but less work on connecting these to supply chain disruptions.^9^ Similarly, policy on extreme events and food security has been predominantly focused on isolated interventions; for example, to deal with acute energy, financial, or trade problems, rather than systemic interventions to improve long-term resilience to multiple kinds of shocks for multiple nations simultaneously.^10^ While the research community does study extreme events and global food security, society continues to experience debilitating impacts on our food systems.

There are two key challenges that present hurdles to effective action. First is the complexity of causation, that is the range of hazards and events that may co-occur and the multiple pathways by which hazards can create societal risks through exposures and vulnerabilities. Second is widespread scientific and political disagreement on the relative efficacy of potential solutions. Both of these factors call for expert elicitation.^11^^,^^12^ Such synthesis could help identify problems and solutions that current data or models may not be able to resolve with an acceptable level of certainty, find areas of consensus, balance viewpoints, and ultimately help both researchers and funding agencies best direct their collective energy and resources to help society tackle these major food system risks.

With ongoing crises affecting food systems—from weather extremes, to COVID-19, to a range of conflicts—a horizon scan and priority-setting exercise is also timely. Such priority-setting exercises have been applied to a range of complex issues, such as economic risks^13^ to linkages between climate change and conflict,^14^ climate resilience,^15^ near term climate impacts,^12^ and conservation,^16^ but not for extreme events and food security.

Individual groups and organizations typically determine their own priorities and research gaps, with the consequence that important interdisciplinary priorities may be overlooked. There is a need to bring together experts in an attempt to build consensus on priorities for research and action to mitigate the effects of extreme events on food insecurity. A key part of this must be identifying major threats and research gaps for both knowledge generation and implementation. Filling such implementation gaps will necessarily require a fuller analysis of trade-offs in policy making, factors influencing adoption of new management practices or technologies, and an assessment of the value of different kinds of knowledge generation, given different capacities for access and utilization across different contexts.

Here, to identify priority food system risks and research opportunities, we surveyed, online and in-person, a group of 69 food system experts (experimental procedures) spanning a range of disciplines and subdisciplines, institutional backgrounds (academia, government and/or international institutions, and NGOs), levels of seniority (e.g., students, postdoctoral researchers, and various levels of faculty), and geographic focus (all continents with permanent human habitation) (Figures S1–S3), on their perceptions of key emerging threats and priority research questions for global food security in the face of extreme events. Our results provide a prioritization of threats to global food security from extreme events, as well as emerging research questions that highlight the conceptual and practical challenges that exist in designing, adopting, and governing resilient food systems. We hope these findings will broadly aid researchers and funders to prioritize their research efforts and focus on the food system transformations needed to deal with the impact of extreme events on global food security.

## Results

### Threat perception

We asked each member of our expert panel to describe a single emerging threat on the horizon—one that they thought would increase global food insecurity in the face of extreme events over the next two decades (the kind of event and threat, e.g., social, biological, political, was left to the discretion of each expert). From 69 submissions, we identified 32 distinct threats, which covered a range of intersecting social, economic, environmental, and geopolitical dimensions. We then asked the experts to rank each threat along two key dimensions—*impact* (the impact on global food security) and *probability* (the probability of occurrence)—following the methods of risk perception commonly used in economic forecasting.^7^ We conditioned average scores on individuals to account for some respondents consistently giving higher or lower scores, a common feature in expert surveys.^11^

We found several cross-cutting themes emerged from the synthesis of the expert elicitation. The first theme, *compounding events and cascading risks*, encompasses both correlated risk of disasters across space and time, sectors, and regions,^17^ as well as specific pathways by which a single hazard can cause a cascade of impacts across food systems.^7^ The second theme, *vulnerability and adaptive capacity*, involves factors that predispose communities to losses, or diminishes their ability to cope with a loss when it occurs.^18^ Finally, *cooperation and conflict*—itself a key component of vulnerability and adaptive capacity—was identified as a third theme, presenting in both acute or chronic conditions, which can undermine communities’ and nations’ abilities to resist and respond to extreme events when they occur.^19^^,^^20^ We explain a selection of such threats below but also include the full list in Table S1.

#### Compounding events and cascading risks

Food system exposure to events of a compound nature has received increasing research attention in recent years^17^ (albeit with a climate focus), so it is perhaps little surprise that our panel identified multiple risks that fell into this category. These included key compound events in specific world regions, such as co-occurring heat waves and droughts in Sub-Saharan Africa^21^ or combined monsoon and meltwater disruptions in Asia.^22^^,^^23^ They also included other globally relevant threats, such as sequential exposure to hazards throughout cropping seasons^24^ or across major breadbaskets,^25^^,^^26^ and co-occurring heat waves at land and sea affecting food supplies.^27^^,^^28^ Physical drivers of these correlated hazards include simple location shifts in temperature distributions across multiple geographies,^29^ disruptions to atmospheric circulation patterns, such as El Niño Southern Oscillation or the North Atlantic Oscillation^30^ and amplified Rossby waves,^31^ as well as the crossing of large-scale tipping points in climate leading to unprecedented weather regimes on a long-term basis.^32^ Exposure of food systems to cascading risks was also of increasing concern and included risk of disruption to critical infrastructure, transport, and public utility systems,^33^ and disruption of choke points in food supply chains impacting multiple processes and actors in food systems simultaneously or in sequence.^34^

#### Vulnerability and adaptive capacity

Like concerns over the changing nature of compound hazards, issues related to vulnerability and adaptive capacity of particular human populations also received attention. Perennial issues, such as increased water demand from population growth—impacting access to clean water, groundwater depletion, and lack of ability to irrigate sustainably—were perceived as top threats that increase vulnerability and reduce adaptive capacity to extreme events.^35^, ^36^, ^37^ Similarly, income reversals for the poor (already happening pre-COVID)^38^^,^^39^ coupled with price transmissions (e.g., global commodity prices causing price spikes in local markets), especially in import-dependent low- and middle-income countries,^40^^,^^41^ were ranked as top threats. Of additional notable concern was an agricultural development trajectory of increasing industrialization leading to a loss of managed diversity on farms (crops and livestock), and concentration of food flows in supply chains and actors^42^^,^^43^; as well as biodiversity loss and loss of ecosystem services supporting food and feed for animal and fish populations.^44^

#### Cooperation and conflict

Finally, human conflict was identified as a key threat to global food security, which could continue to increase over the next two decades. More than 50% of the world’s hungry live in conflict regions, and increasing food insecurity within failed states or in regions with political instability, terrorism, civil unrest, and/or armed conflict was seen as a key threat to global food security by our panel.^2^^,^^45^ Of similar concern were migration and displacement, with associated impacts not only on refugee and migrant food security and nutrition^46^ but also on international cooperation, with important implications for progress on responding to world hunger^20^—a concern supported by recent independent assessments of climate impacts.^12^ Governance failures and geopolitical resource conflict,^47^ resource grabbing on land and sea by wealthier nations that have depleted their own resource bases,^48^ increasing polarization of politics within and between countries, and trade barriers affecting trade and disaster aid^3^ were all also raised as key threats of concern.

#### Other

Other top threats included pest and disease outbreaks, and marine heat waves (one of many emerging threats marine systems face)—both poorly understood issues with the potential to affect large cropland or fisheries areas simultaneously and severely. While fall armyworm and locust outbreaks in Sub-Saharan Africa in recent years have received media attention,^49^ data on pest damage and losses at the field level are poorly documented across the world, with assessments themselves relying on expert elicitation^50^, models built on sparse or coarse resolution data, and simplified assumptions that do not account for the huge diversity of damage functions and interactions between different pests and diseases.^51^^,^^52^ For marine heat waves, only a few experts on our team felt qualified to rank its risk level, but those who did ranked this threat highly, with the importance of this issue being supported by a growing literature on this topic.^53^, ^54^, ^55^

### Research priorities

In addition to asking experts their perceptions on key threats to global food security from extreme events, we also asked participants to identify top-priority research questions on the topic of extreme events and food security. We prioritized the initial 179 responses into 50 by asking the panel to rank the submitted questions along dimensions of research impact and difficulty—how impactful they thought answering the question would be (i.e., in terms of helping to ensure food security in the face of extreme events) and how difficult it would be to answer it (i.e., resources required, time, existence of baseline data and methods, requirements for collaboration across geographies, fields, organizations). Using this prioritization, we differentiated research questions into those that were *lower effort*, i.e., were high-impact research questions but easier to answer from those that were *higher effort*, i.e., high impact but more difficult to tackle. We then grouped the final prioritized questions into three main emergent themes: *better maps and predictions*, *farm-level interventions*, and *food system transformation*, as we explain below.

#### Better maps and predictions

The standard basis for identifying risk, forecasting, and responding to the impact of extreme events on food security is high-quality data. Creating better maps and predictions that can inform proactive prevention and timely response before, during, and after extreme events is crucial. However, the quality, frequency, and spatial extent of validated on-the-ground data on food security have not kept pace with advances in geospatial predictive analytics tools.^56^ Currently, the world’s foremost standard for classifying acute food insecurity, the Integrated Food Security Phase Classification, relies heavily on expert judgment.^57^ Systemic issues relate to limited funding for “boots on the ground” and institutionalized survey programs, poor infrastructure, and low maturity of data governance systems within key nations,^58^ as well as limited programs for grassroots participation in data generation and decision-making on acute and chronic food security. This data availability, access, and utilization problem is exacerbated by logistical challenges that *de facto* accompany extreme events, as seen in conflict zones and with the movement restrictions of COVID-19.^2^^,^^59^ With these critical challenges in mind, which are related to both data generation and the use of data products and services, the lists of questions posed by participants in this category are given in Table 1. These questions clearly highlight frontiers for advanced mapping and analytics and modeling of food systems, while at the same time stressing the need to explicitly monitor, update, and validate the success of these new technologies and insights for improving food security on the ground. While a few are purely methodological, most are thematic.

**Table 1:** Priority research questions on extreme events and food security: Better maps and predictions

Better maps and predictions	lower effort	What are the likely impacts of specific critical infrastructure failures on food security?
		What types of extreme events affect which types of farmers?
		How many individuals are exposed to extreme weather events through hazards which occur in domestic versus export partner countries’ production areas?
		Which import-dependent countries are most vulnerable to climate shocks in major grain exporting countries?
		To what extent can early warning systems identify and inform people most exposed, vulnerable, and unable to adapt to food insecurity challenges in the face of extreme events?
		How can big data, artificial intelligence and machine learning best be used to improve early warning systems?
		How can remote sensing technologies best contribute to reducing food insecurity and better understand increasing extreme events in data-scarce areas?
		How will flooding affect food production and food systems in developing countries in the future?
		Where are the geographic hotspots of food production vulnerability to different kinds of extreme events?
		Which regions of intensive rainfed agriculture will be reliant on irrigation due to extreme reductions in precipitation in the near future?
		Are there tipping points in the intensity of extreme events that will cause global food insufficiency?
		Is the international food trade system dynamic enough to accommodate compound and cascading events?
		Which features of early warning systems are essential for them to be effective?
		What are the likely future impacts of extreme ocean conditions on coastal communities?
	higher effort	How resilient are different food system sectors to a range of key perturbations? (Can they be stress tested?)
		To what extent does early warning for high-risk pest outbreaks for Africa improve food security on the ground?
		How will geographies of pests change in the face of climate change?
		Can we develop reliable globally dynamic predictions of the stocks and flows of food?
		What methods best predict cascading impacts from extreme events across food systems?
		How accurate are food security forecasts across different time scales, and do these forecasts become more accurate by incorporating climate and weather forecasts?
		Can we build accurate sub-seasonal models of precipitation in Sub-Saharan Africa, and what can and cannot be said with the current network of observational weather station data?
		What earth system features (e.g., from the atmosphere, ocean, land surface, and cryosphere) are best at predicting seasonal-scale extremes for key agricultural and populous regions around the globe?
		How does the frequency and intensity of extreme events and their subsequent impact on global food security (from both land and sea) change under different climate change scenarios and shared socio-economic pathways?
		How can artificial intelligence best augment predictions of the probabilities of extreme events and future extreme event occurrence?
		How much would a global network of smart farms providing dynamic data (on farm level soil, water, air, crop changes in response to shocks), help to inform risk reduction for different production systems?

#### Farm-level interventions

A key focal point for research on food security in the face of extreme events is at the farm level. This is because, despite being food producers, many of the world’s farmers, herders, hunters, and fishers, are themselves food insecure. This brings a double benefit to research focused on enhancing resilience to extreme events at the farm level, and in production systems more generally, not only for global food security through stabilizing supply, but also for improved livelihoods. However, food producers operate in socio-ecological systems that may enable or restrict their ability to be resilient or adapt to extreme events. Even when armed with knowledge of resilience-enhancing practices, socioeconomic constraints or incentives can shape vulnerability to extreme events. For example, the presence of well-functioning insurance markets can encourage farmers to plant drought-sensitive crops because of moral hazard (where an actor is incentivized to increase exposure because they do not bear the full costs of that risk),^60^ while the absence of financial markets in combination with liquidity constraints can also prevent farmers from investing in resilient agricultural practices.^61^ One example of the latter mechanism includes slow adoption rates of drought or flood tolerant varieties or irrigation systems in many developing countries.

A range of priority research questions are listed in Table 2. Similar but adapted questions should be explored for populations involved in non-sedentary agriculture, capture and farming fisheries, or hunting and foraging activities. A key overall theme is on farm-level diversification, which is critical given that the dominant agricultural development trajectory has been away from diversified farming systems and toward reduced biodiversity in farming landscapes.

**Table 2:** Priority research questions on extreme events and food security: Farm level interventions

Farm level interventions	lower effort	Which on-farm practices increase resilience to drought, are cost-effective and easily adopted?
		What are the effects of crop diversification on pest, drought, and disease resistance?
		How much can increasing crop diversity improve smallholders’ adaptive capacity?
		How can we best assist food producers in their response to short-term (acute) extreme events (extreme rainfall, high-intensity storms, extreme temperatures, storm surges, etc.)?
	higher effort	How context dependent are on-farm resilience practices across the world, and are there common themes, interventions and technologies that work across multiple locations?
		How does the loss of biodiversity make cropping systems more susceptible to extreme events?
		What are the most effective ways to deploy interventions and increase adoption of on-farm technologies that help reduce the effect of extreme events on food security (e.g., new seed varieties, new irrigation technologies)?

#### Food system transformation

It is widely recognized that system-level interventions are required to address existing structural constraints in food systems,^62^ without which the benefits of better maps and predictions and improved evidence synthesis of farm-level interventions for addressing food security in the face of extreme events will not be realized. Many groups have discussed the issue of transforming food systems for improved resilience, including the Committee on World Food Security at the UN, IPES-Food, International Assessment of Agricultural Knowledge, Science and Technology for Development, and the UN Food Systems Summit (UNFSS).^63^, ^64^, ^65^, ^66^ However, the community currently remains divided on how food system resilience can be increased through the application of specific solutions, with ongoing concerns about the inequitable distribution of power and resources in food systems. The majority of research questions pertaining to this theme (Table 3) were, not surprisingly, deemed more difficult to answer. However, they address many critical issues, which, building on the above categories, sit squarely at the intersection of information generation and availability versus utilization of that information for improving food security. As such, understanding ways to close the implementation gap, with a particular focus on governance, roles of different actors, and the key actions required, underscores a key research priority for improving food security under extreme events.

**Table 3:** Priority research questions on extreme events and food security: Food system transformation

Food system transformation	lower effort	How does crop diversification at the household, community, and regional scales mediate food insecurity during extreme climate events?
		How can food production and supply chains be made robust to disruptions from extreme events affecting multiple regions, or the same region sequentially?
		What climate mitigating steps (e.g., nationally determined contributions, climate-smart agriculture) will also help provide resilience against extreme events?
		What is the effect of agroecological management of food systems on farmer vulnerability to extreme events?
		In what ways does insurance enhance or undermine food security in the face of extreme events?
	higher effort	What does governance for resilient food systems look like?
		What are the major obstacles in developing resilience to extreme events in small-scale farming systems?
		What are the most effective approaches for enhancing adaptive capacities at local and regional levels?
		What are the practical tools and policies for the world’s poor within the scope of limited resources, institutions, infrastructure, capacity, to adapt to extreme events and food insecurity in the near to medium term?
		What are some feasible policy (top down) or community (bottom up) pathways for different sectors to enhance the resilience of food security to extreme events?
		What are policies that make farming systems less vulnerable to extreme events, without negatively affecting other sustainable development goals?
		How does land access affect rural vulnerability to extreme events, and what has been the effect, globally, of land reform efforts on food security and poverty in the face of extreme events?
		What policies are required to ensure that efficiency gains in food distribution systems enable widespread food security without harmimg local and regional producers?
		How can society help establish complex agricultural ecosystems at the farm, watershed, and community levels?
		What are the major barriers undermining the effective uptake of adaptation strategies and how can the limitations associated with these barriers be addressed?
		What are the key societal adaptations required to deal with synchronous crop failures?
		What are the most cost-effective strategies to reduce the impacts of production shocks on food access for the world’s poor?
		What policies prevent extreme events from eroding the capacity of government to protect the food security of citizens?

## Discussion

Identifying key priorities for researchers and funders can be greatly aided through crowdsourcing approaches, which collect the knowledge and wisdom of many, and reduce bias associated with any particular researcher or group.^67^ While similar exercises have been undertaken across a range of fields and topics, this work presents, as far as we know, the first attempt to compile and build consensus on the major threats and priorities for research on food security in the face of extreme events from experts working with diverse backgrounds and expertise and geographic foci. New panel compositions and teams may provide different perspectives, particularly with higher representation of fisheries, livestock, hunting, and foraging expertise. At the same time we recognize that many of the issues we identified are important across these diverse domains. With these points in mind, our results provide some clear insights into some of the major issues threatening global food security from extreme events over the next two decades, as well as examples of some of the top research questions on this topic.

Our analysis found that experts perceived threats on correlated risks across geographies and sequential years to be high. While this topic has previously received attention from a climate perspective,^17^ our analysis extends this further to a broader range of hazards. There was significant concern that compound events will continue to lead to reductions in redundancy, and degrade communities’, regions’, and nations’ abilities to respond to events when they occur. Furthermore, major socio-political, geophysical, and climatological changes in Africa and Asia present key connected and compounding threats to many of the world’s most food insecure.

Our prioritization also indicated that both scientists and practitioners have a need for more granular data and better maps, and improved predictive capacity. New methods of analysis and technologies have allowed for improved advisories, surveillance, monitoring, and humanitarian response,^68^^,^^69^ but at the same time a scarcity of ground data and lack of systems for grassroots data governance, as well as the underutilized role of forecasting in decision-making (such as timely disbursement of resources that limit the scale of disasters), limits the potential of these technologies. There is a need to ensure that the design of new tools, data, and information products is inclusive and coupled with capacity building, improved access and utilization, respect for data sovereignty, and evaluation in terms of ultimate on the ground impact.

Our findings support the notion that the pathway to peace globally remains essential for ensuring global food security in the face of extreme events. Conflict and lack of cooperation—in a variety of manifestations, and at different political scales—continues to present a major impediment to global food security and is a key factor that predisposes communities and nations to disasters following shocks.^3^^,^^19^ Since the inception of this project, civil wars in Syria and Yemen have continued to protract, while several new conflicts have arisen, such as the Ethiopian civil war and the Ukraine-Russia war, all of which threaten regional and global food security.

Markets play an important role in moderating the impacts of local shocks,^66^ through mediating access to resources, incentivizing resilient production practices and spreading risk across geographies. At the same time, markets enhance certain risks that include exposure to risk cascades through interrupted trade and supply chains, price transmission effects for low-income countries, loss of food sovereignty, and redundancy, vulnerabilities that all result from short-term gains inefficiency in food supplies (including “just-in-time” contracting). Markets that fail to price the cost of the loss of food system resilience lead to increased systemic risks. Thus, understanding how to better utilize the power of markets while mitigating the risks they bring to food security is critical to minimize the knock-on effects of extreme events when they do occur.

We found that major threat groupings of conflict, compound/cascading events, and vulnerability and adaptive capacity emerged from our analysis, which raises two interrelated questions: Why did these themes emerge? And what underpins them? Reflecting on this, it can be seen that all carry major uncertainty in terms of effective and timely resolution, all also represent cross-border issues and their resolution requires tackling many outstanding problems of international relations. This uncertainty remains a major challenge. For example, reductions in the likelihood of compound extreme weather events depend on the speed at which governments can collectively realize climate targets. This uncertainty in action on climate change is in turn exacerbated by war and policy responses, including concerns around short-term energy security. Another key connecting thread is that underpins all these emergent categories of risks are failures to engender trust and commit to shared values in international relations and governance.

An important question that arose during our discussions as a group concerned the relative value of prioritizing new research questions, when existing information is not being effectively used for ensuring food security. Part of the gap between knowledge generation and use results directly from access and utilization gaps, themselves representing both hard and soft infrastructure issues, which differentially influence communities' abilities and capacity to access and use information generated by scientists. At the same time, implementation gaps can also exist because of institutional or governance silos, jurisdictional constraints, resource availability constraints, scientific literacy, or political capture by particular actors, which can limit the utilization of information even if it is available and accessible.

Several key research priorities, particularly those from the transformation theme, speak to these issues. We identified questions on enablers of social change, mechanisms for building trust between actors at multiple levels and across contexts, and levers for balancing power and equity in food systems, as well as on developing governance frameworks for ensuring resilience to extreme events. How to translate information and knowledge into action is clearly itself a key research priority and represents a large knowledge gap. These research questions are highly complex, demand strong multidisciplinary expertise and approaches, and require new funding efforts and coordination to assess which kinds of information hold the most value for leveraging change. They also require researchers to step beyond their own silos and place their efforts where they are most needed to enable such transformations.

Extreme events impact global food security through a multitude of pathways. Some of the threats highlighted here are recognized by the panel to be “already happening” or “age-old issues,” but are of magnified importance in the next two decades. In contrast, some of the complex linkages between social and natural systems identified here in the context of extreme events are only just beginning to be made by others.^70^ There is little doubt that COVID-19 and ongoing and emerging conflicts have shed new light on these kinds of problems. We are all mindful that research and resources in the public and private sectors are not infinite; at the same time, tackling some of the highest impact research questions will require significant investments of time and money. However, these investments are needed, as extreme events will continue to threaten global food security over the near to medium term. As such, we see it as our responsibility, as practitioners and researchers with expertise in this area, to join forces and help address these challenges head-on.

## Experimental procedures

### Resource availability

#### Lead contact

Further information and requests for resources should be directed to and will be fulfilled by the lead contact, Zia Mehrabi (zia.mehrabi@colorado.edu).

#### Materials availability

No new materials were generated in this study.

### Initial surveys

We used a modified version of the Delphi technique^71^ to identify threats for extreme events and food security globally. This horizon scanning method has been applied successfully in a similar manner to identify emerging issues for global conservation and biological diversity.^16^ We also used expert elicitation and a priority-setting exercise to identify priority research questions for extreme events and food security. This method, or variations of it, have been applied to identify top research questions in a range of research fields and contexts.^72^

We surveyed experts on top threats and priority research questions by (1) circulating a request to participate within our professional networks with an initial online survey and, following this (2) an in person workshop session on “Extreme Events and Food Security” at the “Extreme Events—Building Climate Resilient Societies” conference, which was funded by the VW Foundation and held over November 9–11, 2019 in Hannover, Germany. We asked for open-format text answers to the following question:

Describe an emerging threat on the horizon, which could increase food insecurity in the face of extreme events over the next two decades.

Participants were given 100 words to describe one threat and asked to add references and supporting sources. As part of these same initial submission rounds, we also asked participants to identify up to three responses to the question:

Identify a top priority research question on the topic of extreme events and food security.

In the first round of expert elicitation via the online survey and workshop, we received 69 replies (69 threats, 179 questions) from participants covering a wide range of expertise, institutional background (academia, government, or supranational institutions, and NGOs), seniority (Ph.D. students, PostDocs, and various levels of Professors and Lecturers), and geographic focus (all continents were covered, with a particular emphasis on Africa and Asia). This experience can be seen in Figures S1–S3, which show the details of participants’ area of expertise, years of experience, and geographic research focus as collected on the initial online survey.

### Workshop

During the in-person workshop, we collected all the online and in person submissions and undertook a pilot ranking exercise of the responses. Here, we separated the threats and questions into preliminary themes and asked two to three experts that self-identified with each theme to review the list, undertake a pilot ranking exercise to test the prioritization methodology, to identify any extensively broad or duplicate questions that should be eliminated and to make any other suggestions.

After the workshop, a moderator removed threats and questions flagged as obvious duplicates or being too broad, and reworded others for clarity, resulting in 32 threats and 147 questions that were sent out to the full group of contributors in a second online survey. We did not remove “low-quality research questions.”^73^ While we recognize this may have resulted in some questions being of perceived higher research quality than others (e.g., having both theoretical and empirical components, sufficient granularity, not being double barrelled), we wished to maintain, as much as possible, the diversity of different ideas and sources submitted for full consultation.

### Prioritization

We compiled these revised lists into a second set of online surveys for prioritization. We invited the full list of experts (n = 69), to rank the list of refined threats and research questions, each presented in their own survey, in which items were presented in randomized order per participant. Participants were asked to rank each item (whether a threat of research question) on simple Likert scales (high to low; 1 to 5, with non-anchored intervals). For threats, these scales included *impact* (What is the impact of this threat on global food security?) and *probability* (What is the probability of this threat occurring?). For research questions, these scales included *impact* (How much impact do you think the research question will have if answered?), *difficulty* (What difficulty level does this research question have?), and *expertise* (What is your level of expertise in the topic area of this question?). In total, we received n = 30 and n = 29 responses for threat and research question prioritization surveys, respectively. We piloted each survey before sending it out to confirm the estimated time for the survey, and to ensure it was navigable and that the FAQ was clear.

We then used hierarchical cumulative link models^74^ to estimate the modes of the Likert scales, and probabilities of those modes, for each threat and research question, and for each response (e.g., *impact*, *probability* for threats, and *impact* and *difficulty* for research questions) conditioned on individuals (which were treated as random intercepts). For research questions we also conditioned mode probability estimates on expertise level (treated as fixed intercepts) to account for higher likelihood of individuals giving higher priority for questions related to their own fields.

We then ranked each outcome on the concatenation of the mode and probability (e.g., if for *impact* a selection of threats had modes of 5, 5, 4, and probabilities of those modes 0.8, 0.6, 0.9, their concatenation would be 5.08, 5.06, 4.09; and we would rank them in order 1, 2, 3 from most to least impactful). For identifying top threats (most impactful and highest probability of occurrence) we simply computed the mean rank of *impact* and *probability* ranks, and ranked those mean ranks (these ranks are shown in Table S1). For identifying top research questions, we identified the top-ranking 50 questions in terms of *impact* and then split them into higher- and lower-hanging questions based on a simple percentile split in ranks of *difficulty*.

We then collated the final threats and research questions into emergent themes post prioritization and added examples into a first manuscript draft. We shared this manuscript draft with the full list of contributing experts (n= 69) for review and allowed experts to submit suggestions and thoughts, as well as textual edits to the final lists.

## Data Availability

Anonymized versions of data and scripts can be accessed at https://doi.org/10.5281/zenodo.6785659.

## Supplemental information

Document S1. Figures S1–S3 and Table S1

Document S2. Article plus supplemental information
