# Supplementary material for: Research priorities for global food security under extreme events
Source: One Earth. 2022 Jul 15;5(7):756–66. doi: 10.1016/j.oneear.2022.06.008 (PMC9307291; doi:10.1016/j.oneear.2022.06.008)
Supplement: Document S1. Figures S1–S3 and Table S1 [file mmc1.pdf]

One Earth, Volume *5*

Supplemental information

Research priorities for global food security under extreme events

Zia Mehrabi, Ruth Delzeit, Adriana Ignaciuk, Christian Levers, Ginni Braich, Kushank Bajaj, Araba Amo-Aidoo, Weston Anderson, Roland A. Balgah, Tim G. Benton, Martin M. Chari, Erle C. Ellis, Narcisse Z. Gahi, Franziska Gaupp, Lucas A. Garibaldi, James S. Gerber, Cecile M. Godde, Ingo Grass, Tobias Heimann, Mark Hirons, Gerrit Hoogenboom, Meha Jain, Dana James, David Makowski, Blessing Masamha, Sisi Meng, Sathaporn Monprapussorn, Daniel Müller, Andrew Nelson, Nathaniel K. Newlands, Frederik Noack, MaryLucy Oronje, Colin Raymond, Markus Reichstein, Loren H. Rieseberg, Jose M. Rodriguez-Llanes, Todd Rosenstock, Pedram Rowhani, Ali Sarhadi, Ralf Seppelt, Balsher S. Sidhu, Sieglinde Snapp, Tammara Soma, Adam H. Sparks, Louise Teh, Michelle Tigchelaar, Martha M. Vogel, Paul C. West, Hannah Wittman, and Liangzhi You

One Earth, Volume 5

Supplemental information
Research priorities for global food security under extreme events

Zia Mehrabi, Ruth Delzeit, Adriana Ignaciuk, Christian Levers, Ginni Braich, Kushank Bajaj, Araba Amo-Aidoo, Weston Anderson, Roland A. Balgah, Tim G. Benton, Martin M. Chari, Erle C. Ellis, Narcisse Z. Gahi, Franziska Gaupp, Lucas A. Garibaldi, James S. Gerber, Cecile M. Godde, Ingo Grass, Tobias Heimann, Mark Hirons, Gerrit Hoogenboom, Meha Jain, Dana James, David Makowski, Blessing Masamha, Sisi Meng, Sathaporn Monprapussorn, Daniel Müller, Andrew Nelson, Nathaniel K. Newlands, Frederik Noack, MaryLucy Oronje, Colin Raymond, Markus Reichstein, Loren H. Rieseberg, Jose M. Rodriguez-Llanes, Todd Rosenstock, Pedram Rowhani, Ali Sarhadi, Ralf Seppelt, Balsher S. Sidhu, Sieglinde Snapp, Tammara Soma, Adam H. Sparks, Louise Teh, Michelle Tigchelaar, Martha M. Vogel, Paul C. West, Hannah Wittman, and Liangzhi You

Supplemental Figures

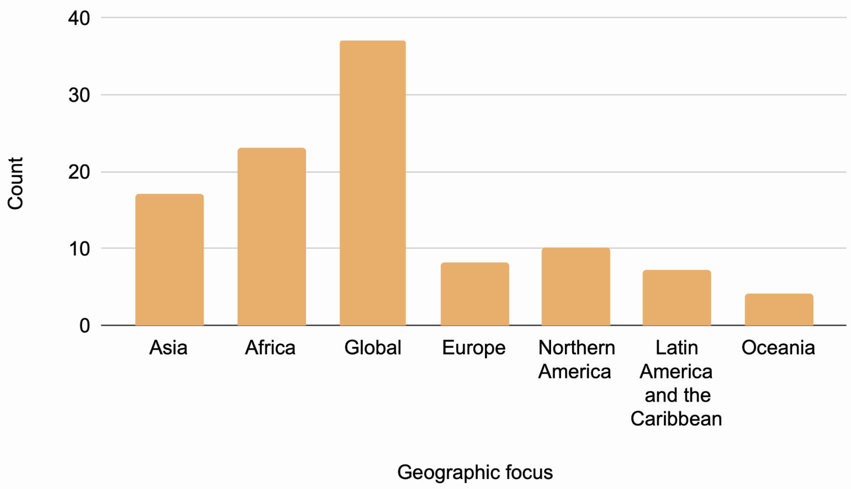

Figure S1 Geographic expertise of experts contributing to the study. Note, experts were allowed to declare more than one focal geography. Data were collected in the initial online survey (see Experimental Procedures).

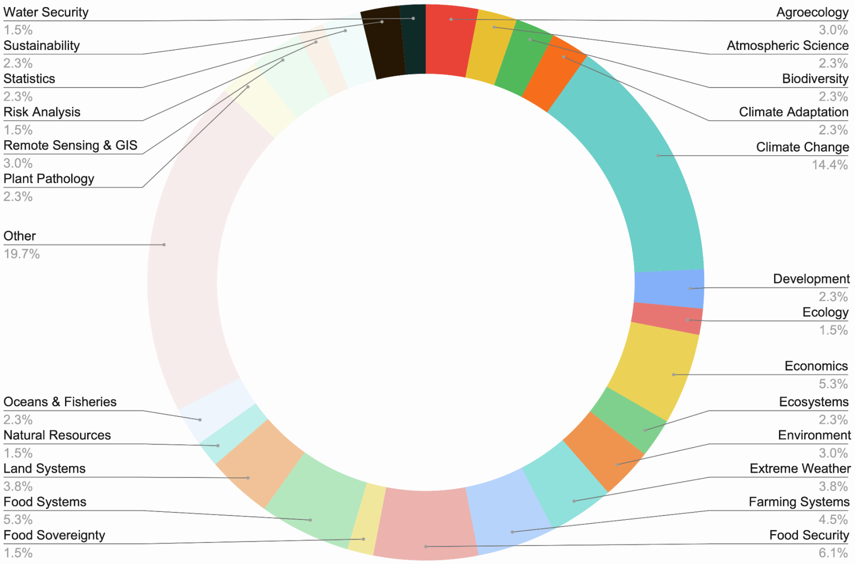

Fig S2 Field of expertise from experts contributing to the study. Note, these were self declared, and some experts declared more than one area of expertise. “Other” includes a range of of additional experts foci not shown, in philosophy, ethics, rural sociology, conflict science, humanitarian response, plant genomics, livestock systems, crop modeling, coastal hazards, environmental monitoring, drought management, urban food systems, gender analysis, landscape ecology, phytosanitation, human nutrition, socio-ecological systems, enterprise management, research performance evaluation, and impact assessment. Data were collected in the initial online survey.

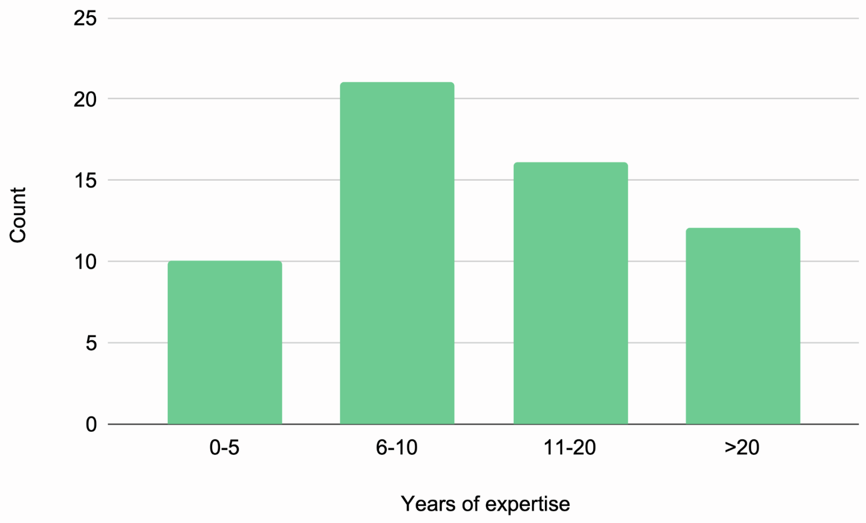

Fig S3 Years of expertise in the declared field from experts contributing to the study. Data were collected in the initial online survey.

Supplemental Tables

Table S1. List of threats ranked and prioritized in this study. See main text for additional context and discussion.

Title	Class	Text	Rank (Impact)	Rank (Probability)	Mean Rank
Increased water demand	Vulnerability/ adaptive capacity	Combination of rising water demand as well as low innovation in ways of growing food with limited amounts of water, will lead to further water insecurity in the face of climate extremes, particularly in irrigation dependent production systems, which will be amplified by population growth, urbanization, and the over-reliance on non-renewable resources, especially groundwater.	1	2	1.5
Drought & heat waves in SSA	Compound events	Losses to crop production by droughts and heat waves in Sub-Saharan Africa resulting in significant increases in food insecurity in the region.	2	1	1.5
Collapse of ecosystem services	Vulnerability/ adaptive capacity	The co-occurrence of extreme events, biodiversity loss, and ecosystem service collapse with negative effects on food production, food prices, and ultimately food security, through loss of essential services such as water regulation, pollination and pest control, and supporting food and feed for fish and animal populations.	4	6	5
Marine heat waves	Other	Heat waves and other extreme events negatively impacting marine resources through changes in their abundance and distribution, especially impacting coastal systems, and dependent communities in small and low income countries.	3	8	5.5
Income inequality	Vulnerability/ adaptive capacity	Production losses and associated price spikes not accompanied by rapid income growth for the poor putting the most vulnerable communities at even greater risk to food insecurity through increased poverty limited access.	12	3	7.5
Political instability and migration	Co-operation/ conflict	Extremes events amplifiying food insecurity from, as well as increasing, conflict, terrorism, and migration/displacement within and between nations.	11	4	7.5

Title	Class	Text	Rank (Impact)	Rank (Probability)	Mean Rank
Pest and disease outbreaks	Other	More frequent and severe weather, combined with long term climate change impacts on novel pest distriutions, will lead to increasing pest pressure, more severe outbreaks, and a breakdown in genetic resistance, which will result in significant crop losses and health threats for humans and animals.	10	7	8.5
Monsoon & meltwater disruption in Asia	Compound events	Major disruptions of monsoon patterns and alterations of meltwater flow patterns in major river basins negatively affecting agricultural production due to missing irrigation water in Asia, and impeding food security for billions dependent on these water resources.	8	10	9
Price shocks and volatility	Vulnerability/ adaptive capacity	Extreme events inducing global food price shocks, which will affect middle and low income countries the most. The strong global market integration of these countries make them vulnerable for price fluctuations transmitted to their local markets and oftentimes these countries lack the capacity to protect their local markets (e.g. because of trade agreements, lack of storage facilities).	14	5	9.5
Low agricultural diversity	Vulnerability/ adaptive capacity	An increasing simplification of global agricultural systems through monoculture cropping and livestock genetics, will make these systems highly dependent on agrochemical inputs and more vulnerable to a range of climatic risks, evolution of pesticide resistance, fuel price volatility, and epidemics.	16	9	12.5
Climate tipping points	Compound events	The crossing of large-scale tipping points in climate will lead to fundamentally different climate regimes and unprecedented weather regimes on a long-term basis. Exceeding those tipping points will have also negative feedback effects by accelerating and intensifying climate change and extreme weather events.	5	20	12.5

Title	Class	Text	Rank (Impact)	Rank (Probability)	Mean Rank
Adaptive tipping points	Compound events	An increase in extreme events frequency and severity leading to continued and time compounded losses to agricultural productivity across sequential cropping cycles, exacerbating and accelerating impacts of individual events, and reducing farm level resiliency and adaptive capacity.	13	17	15
Unpredictable weather changes	Other	Major shifts in weather patterns such as storms and rainfall and temperature extremes disproportionally affecting rural communities. Aggravated by changes in climate teleconnection patterns, rendering existing agricultural knowledge of seasonality less useful.	17	14	15.5
Compound heat waves on land	Compound events	Compound heat waves in space and/or time will aggravate individual heat-related impacts on food production. Simultaneous production shocks from multiple heat waves across agricultural regions have the potential to increase global food prices and food insecurity.	9	24	16.5
Breadbasket failure	Compound events	Multiple breadbasket failures, resulting from co-occuring climate extreme events, pests, and diseases as well as the lack of buffering capacity of global markets, will lead to long-term stability of food and nutrient provisioning.	7	26	16.5
Breeding failures	Vulnerability/ adaptive capacity	Difficulties to breed tolerance to heat stress because of physiological constraints and because the interaction of genetics and environmental factors on plant responses under extremely high temperatures is largely unknown.	22	12	17
Compound heat waves on land and sea	Compound events	Co-occurring heat waves on land and sea as the result of shifting mean climates and higher probability of extreme land and sea temperatures leading to both loss of crop yields and available fish catch, leading to a double whammy of food supply shortages.	6	29	17.5
Resource conflict	Co-operation/ conflict	Resource grabbing on land and sea by powerful countries that have exploited their own resource base, and governance failures to control this activity, amplifying the impact of extreme events for the most vulnerable by reducing their capacity to grow, hunt, or access food.	24	11	17.5

Title	Class	Text	Rank (Impact)	Rank (Probability)	Mean Rank
Trade barriers	Co-operation/ conflict	The increasing number and strength of trade barriers by many industrialized and BRIC countries affecting both open trade and disaster aid needed for resilience to shocks to major breadbaskets failures due to extreme events.	21	15	18
Increase in civil unrest	Co-operation/ conflict	Production losses and reduced resource bases and rising food prices as the result of extreme events increasing riots, civil unrest and armed conflict, especially in failed/ unstable states.	18	19	18.5
Loss of subsistence capacity	Vulnerability/ adaptive capacity	The interplay between the scale transition to less farmers operating larger farms and reduction in subsistence farming, with increased market dependency for food, will lead to high exposure and food insecurity in the face of extreme events, especially for underprivileged and poorer communities.	25	13	19
Loss of food sovereignty	Vulnerability/ adaptive capacity	The continued rise in corporate control of the food system and the inability to institutionalize and enforce The Right to Food will severely affect the livelihoods of low-income communities and hinder their access to healthy food in the face of extreme events.	23	16	19.5
Critical infrastructure disruption	Compound events	Damage to critical infrastructure and public utility systems, leaving millions of households affected by minor inconveniences (such as power outages of short duration) to more severe disruptions (such as extended loss of utilities and public services for days and weeks, and the long-term shut-down of bridges, roads, and other transportation networks), with significant disruptive impacts on food insecurity.	19	23	21
Multiple supply chain failures	Compound events	The correlated risk of extreme events throughout supply chains leading to simultaneous stressors on the production, stocking, transport, storage, and retail components of agricultural systems. This is particularly problematic if 'choke points' are affected.	15	30	22.5
Climate skepticism	Co-operation/ conflict	An increase in climate skepticism hindering timely and effective implementation of adaption and mitigation strategies.	20	25	22.5

Title	Class	Text	Rank (Impact)	Rank (Probability)	Mean Rank
Workforce heat stress	Other	Extreme heat and other climatic factors having adverse health impacts on farmers and crop workers, and negatively impacting food security both through productivity losses, and for the workers themselves through income loss or health detriments (e.g. from heat exposure or nocturnal working hours) .	32	18	25
Ageing farming populations	Vulnerability/ adaptive capacity	A growing age of farmers in agriculture and the lack of successors from younger generations creating severe difficulties for adaption to extreme events.	29	22	25.5
Agricultural intensification	Vulnerability/ adaptive capacity	Global trends of intensifying agricultural systems by conventional means (i.e. optimized for increased yields and calories) further increasing their susceptibility to climate extreme events.	31	21	26
Loss of human co-operation	Co-operation/ conflict	Further polarization of politics across a range of scales will lead to increasingly competitive rather than collaborative forms of governance between communities and countries, undermining co-operation at different levels in society.	27	28	27.5
Increased gender inequality	Vulnerability/ adaptive capacity	Extreme events leading to exacerbation of existing gender inequality, which will entail substantial negative impacts for food security given womens key roles in agricultural production, and food provision within households.	28	27	27.5
Destabilization of pollution sources	Compound events	Threats from nuclear or other major industrial/pollution sources that are susceptible to extreme events severely damaging terrestrial, marine, and other aquatic resources simultaneously.	26	32	29
Agricultural investment failures	Vulnerability/ adaptive capacity	Diminished agricultural investments resulting in negative consequences for the creation of globally sustainable and resilient food systems.	30	31	30.5
